# In Search of the Biological Significance of Modular Structures in Protein Networks

**DOI:** 10.1371/journal.pcbi.0030107

**Published:** 2007-06-01

**Authors:** Zhi Wang, Jianzhi Zhang

**Affiliations:** Department of Ecology and Evolutionary Biology, University of Michigan, Ann Arbor, Michigan, United States of America; University of California San Diego, United States of America

## Abstract

Many complex networks such as computer and social networks exhibit modular structures, where links between nodes are much denser within modules than between modules. It is widely believed that cellular networks are also modular, reflecting the relative independence and coherence of different functional units in a cell. While many authors have claimed that observations from the yeast protein–protein interaction (PPI) network support the above hypothesis, the observed structural modularity may be an artifact because the current PPI data include interactions inferred from protein complexes through approaches that create modules (e.g., assigning pairwise interactions among all proteins in a complex). Here we analyze the yeast PPI network including protein complexes (PIC network) and excluding complexes (PEC network). We find that both PIC and PEC networks show a significantly greater structural modularity than that of randomly rewired networks. Nonetheless, there is little evidence that the structural modules correspond to functional units, particularly in the PEC network. More disturbingly, there is no evolutionary conservation among yeast, fly, and nematode modules at either the whole-module or protein-pair level. Neither is there a correlation between the evolutionary or phylogenetic conservation of a protein and the extent of its participation in various modules. Using computer simulation, we demonstrate that a higher-than-expected modularity can arise during network growth through a simple model of gene duplication, without natural selection for modularity. Taken together, our results suggest the intriguing possibility that the structural modules in the PPI network originated as an evolutionary byproduct without biological significance.

## Author Summary

Many complex networks are naturally divided into communities or modules, where links within modules are much denser than those across modules. For example, human individuals belonging to the same ethnic groups interact more than those from different ethnic groups. Cellular functions are also organized in a highly modular manner, where each module is a discrete object composed of a group of tightly linked components and performs a relatively independent task. It is interesting to ask whether this modularity in cellular function arises from modularity in molecular interaction networks such as the transcriptional regulatory network and protein–protein interaction (PPI) network. We analyze the yeast PPI network and show that it is indeed significantly more modular than randomly rewired networks. However, we find little evidence that the structural modules correspond to functional units. We also fail to observe any evolutionary conservation among yeast, fly, and nematode PPI modules. We then show by computer simulation that modular structures can arise during network growth via a simple model of gene duplication, without natural selection for modularity. Thus, it appears that the structural modules in the PPI network may have originated as an evolutionary byproduct without much biological significance.

## Introduction

**Figure 1:**
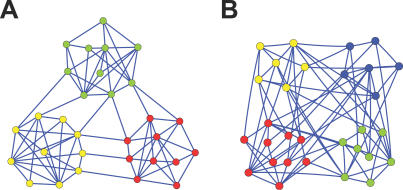
An Example of a Small Network with a Modular Structure (A) and Its Randomly Rewired Network (B) Different colors show different modules separated by Guimera and Amaral's algorithm [28]. The modularity is 0.5444 for the network in (A) and 0.2838 in (B), and the scaled modularity is 15 for the network in (A) and 0 in (B).

**Figure 2:**
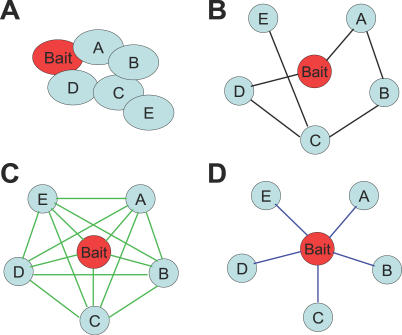
PPI Network Representations of Protein Complexes (A) A hypothetical protein complex. Binary protein−protein interactions are depicted by direct contacts between proteins. Although five proteins (A, B, C, D, and E) are identified through the use of a bait protein (red), only A and D directly bind to the bait. (B) The true PPI network topology of the protein complex. (C) The PPI network topology of the protein complex inferred by the “matrix” model, where all proteins in a complex are assumed to interact with each other. (D) The PPI network topology of the protein complex inferred by the “spoke” model, where all proteins in a complex are assumed to interact with the bait; no other interactions are allowed.

Many complex networks are naturally divided into communities or modules, where links within modules are much denser than those across modules [1] (Figure 1). For example, human individuals belonging to the same ethnic groups interact more than those from different ethnic groups [2]. Studying the modularity of a network not only provides structural information about the network, but may also reveal the underlying mechanisms that determine the network structure. The concept of modularity is not new to biologists. In fact, cellular functions are widely believed to be organized in a highly modular manner, where each module is a discrete object composed of a group of tightly linked components and performs a relatively independent task [3–7]. It is interesting to examine whether this modularity in cellular function arises from modularity in molecular interaction networks such as the transcriptional regulatory network and protein–protein interaction (PPI) network. Many authors have attempted to separate modules in the PPI network based on either the network topology alone or with additional information about gene function and expression [8–16]. They generally report high modularity in the PPI network, with evidence for a rough correspondence between PPI modules and functional units. All these analyses, however, suffered from a serious bias in the current PPI data. The PPI data include binary interaction information that is either directly obtained from experiments such as the yeast two-hybrid (Y2H) assay [17,18], or indirectly inferred from stable protein complexes [19]. High-throughput protein complex identification is usually mass-spectrometry–based [20–23] (e.g., tandem-affinity purification). These methods involve the discovery of a complex of interacting proteins including a tagged bait protein, but do not provide information about direct pairwise protein–protein interactions [19,24]. Some small-scale biochemical methods, such as co-immunoprecipitation [25] and affinity precipitation [26], can also identify protein complexes without providing pairwise protein interaction information. Protein complex data obtained by one of these methods are then translated into binary PPIs by either the “matrix” or the “spoke” model [19] (Figure 2). The matrix model assumes that all members of a protein complex interact with each other, whereas the spoke model assumes that all nonbait members of a complex interact with the bait. It is obvious that use of the matrix model creates PPI modules corresponding to protein complexes. The spoke model can also affect modularity because the bait is interpreted by the model as a hub (i.e., a highly connected node), while in reality it may not be a hub. Because the reliability of the two models is unknown, it is possible that the prevailing modularity of PPI networks is an artifact of these models. In this work, we explore the above possibility by analyzing the modularity of two yeast PPI networks. The first is referred to as the PIC network, as it is the PPI network including protein complex data, whereas the second is named the PEC network, as it the PPI network excluding all edges inferred from protein complexes. Because we are assessing the modularity of the PPI network per se, only the network topology will be used in separating modules. Our analyses show that although both PIC and PEC networks are highly modular, the identified modules lack obvious correspondence to functional units and are not evolutionarily conserved. We use computer simulation to show that modularity can arise in a simple model of network growth through gene duplication, without the involvement of selection for modularity. Together, our findings suggest that structural modules in PPI networks may have arisen as an evolutionary byproduct without biological significance.

## Results

### Do PPI Networks Show Modular Structures?

We downloaded the PPI data for the budding yeast Saccharomyces cerevisiae from the Munich Information Center for Protein Sequences (MIPS) [27]. The dataset was human-curated and contained mostly binary interactions directly observed in Y2H experiments. In addition, about 10% of the binary interactions in the dataset were inferred using either the spoke or matrix model from protein complexes identified by high-confidence small-scale experiments. This entire dataset is referred to as the PIC network here. Based on the MIPS annotation, we removed from the PIC network those binary interactions that were inferred from protein complexes, resulting in the PEC network. Because it is only meaningful to separate modules within a connected part of a network, we studied the largest connected subset (i.e., the giant component), of a network. The giant component contains more than 90% of all nodes in the yeast PPI network. For simplicity, we refer to the giant component of a network as the network, unless otherwise noted. Table 1 lists some important parameters for the PIC and PEC networks studied here.

**Table 1:**
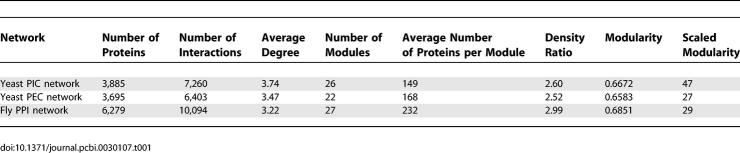
Summary Statistics of the Giant Component of the Protein Interaction Networks

The extent of modularity for a particular modular separation of a network is often measured by *M* = 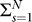 [(*l_s_*/*L*) − (*k_s_*/2*L*)^2^], where *N* is the number of modules, *L* is the total number of edges in the network, *l_s_* is the number of edges within module *s*, and *k_s_* is the sum of the degrees of the nodes in module *s* [28,29]. The degree of a node is simply the number of edges that the node has. The particular separation that maximizes *M* is considered the optimal modular separation and the corresponding *M* is referred to as the modularity of the network (Figure 1). In essence, *M* is the difference between the observed and expected proportions of within-module edges in the network. Here, the expected proportion is computed from a nonmodular network where edges are equally likely to be within and between modules.

Several algorithms are available to separate a network into modules and obtain the maximal *M*. Empirical and simulation studies showed that the method of Guimera and Amaral [28] has the best performance because it can give the most accurate module separation and highest *M* [30]. We therefore used this method to separate modules in the yeast PIC and PEC networks. To obtain the highest *M*, we used delicate parameter settings in the simulated annealing algorithm. It took a typical desktop computer ~3 d to separate a yeast PPI network. The PIC network is separated into 26 modules with a modularity of 0.6672, while the PEC network is divided into 22 modules with a modularity of 0.6583 (Table 1). The density ratio, defined by the ratio of the number of within-module edges to the number of between-module edges, is only slightly lower for PEC than for PIC networks (Table 1).

**Figure 3:**
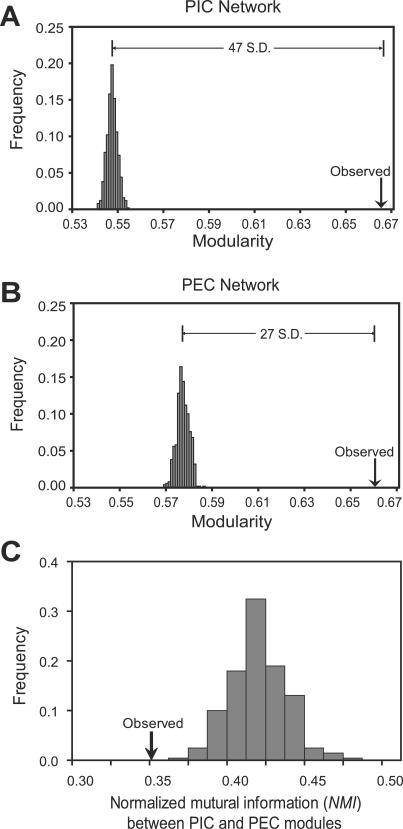
The Modularity of Yeast PIC and PEC Networks Compared with That of Their Randomly Rewired Networks, and the Similarity of Module Compositions between PIC and PEC Networks Compared with the Random Expectation (A,B) The observed modularity is indicated by the vertical arrow. The bars show the frequency distribution of the modularity from 500 randomly rewired networks. Scaled modularity, or the difference between the modularity of a real network and the expected modularity of a randomly rewired network in terms of the number of standard deviations, is indicated at the top area of the panel. (C) The observed similarity between PIC and PEC networks, measured by *NMI,* is indicated by the vertical arrow. The bars show the frequency distribution of the *NMI* between PIC and 200 reduced networks (by random removal of 10% edges from the PIC network). The result shows that the difference between PEC and PIC is not simply because the PEC network is 10% smaller than the PIC network.

A random network may also have a nonzero modularity by chance or due to certain degree distributions [31]. Also, the modularity values of two networks with different sizes or different average degrees cannot be compared directly [31]. Thus, to measure the modularity of a network, we compare it with a random network of the same size and same degree distribution, which is generated by the local rewiring algorithm [32]. To speed up the computation, we used moderate parameter settings and faster runs (~4 h per network) to estimate modularity. For the yeast PIC network, the modularity for 500 randomly rewired networks has a mean of 0.5466 and a standard deviation of 0.0023, while the real PIC network has a modularity of 0.6555 under this parameter setting (Figure 3A). We use z-score, or the number of standard deviations higher than the random expectation to measure the deviation of the modularity of a network from its random expectation. This z-score, referred to as the scaled modularity to differentiate it from z-scores of other properties, is (0.6555 – 0.5466)/0.0023 = 47 for the PIC network. Under the same parameter setting, the modularity for the real PEC network is 0.6481. The modularity for 500 randomly rewired PEC networks has a mean of 0.5764 and a standard deviation of 0.0027 (Figure 3B). In other words, the scaled modularity for the PEC network is (0.6481 − 0.5764)/0.0027 = 27. Thus, both PIC and PEC networks show significantly greater modularity than randomly rewired networks. As expected, the scaled modularity of PIC is much greater than that of PEC. This difference is largely due to the exclusion of protein complex data in the PEC network. In fact, when we randomly removed 10% of edges from the PIC network, the scaled modularity decreased only slightly (from 47 to 42).

Given the substantive difference in scaled modularity, PIC and PEC networks should also differ in the compositions of their modules. We measured the similarity in module composition between different separations of the same network (or shared nodes in the case of different networks) by the normalized mutual information *(NMI)* index [30]. A higher *NMI* indicates a higher similarity in module composition. The *NMI* between the PIC network and PEC network is 0.35. As a control, we measured the *NMI* between the PIC network and a reduced network generated by random removal of 10% of the edges in PIC. This control *NMI* has a mean of 0.41 and a standard deviation of 0.018 (from 200 replications). Thus, the *NMI* between PIC and PEC is significantly lower than that between PIC and its randomly reduced networks (*p* < 0.002) (Figure 3C). Because simulated annealing is a stochastic algorithm, different runs may yield slightly different partitions. We thus separated modules in PIC and PEC networks with different random seeds 50 times, and these replications confirmed that the above finding of a lower *NMI* between PIC and PEC than by chance is genuine (*p* < 10^−10^, Mann-Whitney U test). Together, these analyses demonstrate that the inclusion of interactions inferred from protein complexes in the PPI network has a great impact on network modularity.

### Are Structural Modules Functional Units?

**Figure 4:**
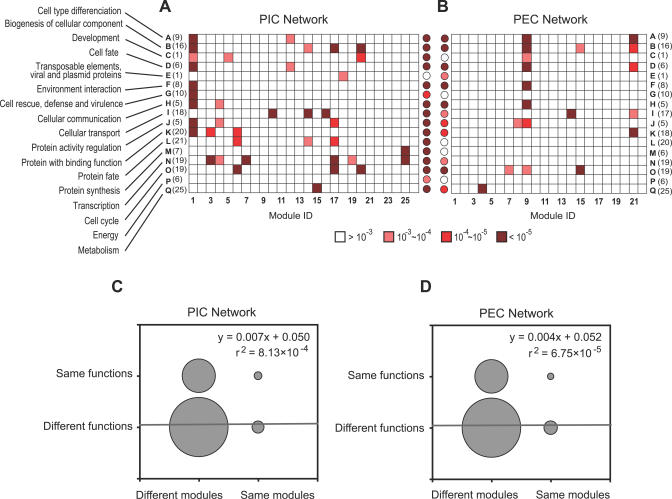
Lack of Obvious Correspondence between Structural Modules and Functional Units (A,B) Each functional category is indicated by a letter (A to Q). In parentheses next to the letter is the percentage of proteins in the network that belong to that functional category. Note that one protein may belong to more than one category. The circles next to the grid show the statistical significance of nonrandom distributions of genes of the same functional categories across modules. Each small square in the grid shows the statistical significance of enrichment of a particular function in a module. For the circles and squares, significance levels are indicated by different colors. (C,D) Show the correlation between co-membership in structural modules and co-functionality for all pairs of proteins in the PIC and PEC networks, respectively. The circle size is proportional to the number of protein pairs. The line shows the linear regression and *r* is the correlation coefficient.

Because we identified the PPI modules based entirely on the topology of the network, it is important to ask whether such structural modules correspond to functional units. To address this question, we utilized the functional annotation of yeast genes in the CYGD database [33]. At the highest level of annotation, each yeast gene is classified into one or several of 17 functional categories (Figure 4). If the structural modules correspond to functional units, we should expect a nonrandom among-module distribution of the genes of a given functional category. For example, in the PIC network, there are 361 genes belonging to functional category A (cell type differentiation; see Figure 4A). A χ^2^ test showed that these genes are not randomly distributed across the 26 PIC modules (χ^2^ = 317, df = 25, *p* < 10^−5^; see the circles in Figure 4A). This test was conducted for each functional category, and almost all functional categories showed significant nonrandom distributions across PIC modules (even after considering multiple testing). In contrast, the PEC network has fewer functional categories showing significant nonrandom distributions. This trend is particularly evident at the highest level of statistical significance (six categories in PEC versus 14 in PIC) (Figure 4B).

If structural modules correspond to functional units, we also expect that the majority of genes in a module belong to only one or a few functional categories. In other words, each module should have one or a small number of overrepresented functional categories. Testing this prediction is not easy because one gene may belong to multiple functional categories. We thus used computer simulations. For example, module 1 of the PIC network comprises 227 proteins, 92 of which belong to functional category A (Figure 4A). We randomly chose 227 genes from the network and counted the number of category A genes. We repeated this procedure 100,000 times to estimate the probability that the number of category A genes in the randomly picked 227 genes is equal to or greater than 92. This probability is indicated with different colors in the small squares of Figure 4A. Because 17 functional categories were tested for each module, to control for multiple testing we used 10^−3^ as the cutoff for statistical significance for each category. It can be seen that in 16 (62%) of the 26 PIC modules, at least one functional category is enriched. In comparison, only 7 (32%) of the 22 PEC modules have at least one enriched functional category. The above difference between PIC and PEC modules is statistically significant (*p* < 0.05, χ^2^ test).

The two analyses above revealed nonrandom distributions of protein functions across structural modules. To quantitatively measure how well structural modules correspond to functional units, we used a correlation analysis. For a pair of proteins from a PPI network, we ask if they belong to the same module (co-membership) and if they belong to the same functional category (co-functionality). Two proteins are considered to possess co-functionality as long as they share at least one function. If structural modules correspond well to functional units, protein pairs within the same module should share function whereas protein pairs across modules should not share function. In other words, we should observe a strong positive correlation between co-membership and co-functionality of protein pairs. We enumerated all possible protein pairs and found the correlation to be statistically significant in both PIC (*p* < 10^−300^) and PEC (*p* < 10^−100^) networks. However, the level of correlation is extremely low in both PIC (*r^2^* = 0.0813%) and PEC (*r^2^* = 0.00675%) networks (Figure 4C and 4D), indicating that less than 0.1% of the variance in protein-pair co-membership is explainable by co-functionality. We also found that the *r* value for PEC is significantly lower than that for PIC when we repeated module separations 50 times with different random seeds (*p* < 10^−5^, Mann-Whitney U test). The observation of a low level of correlation is not due to the presence of many multifunctional proteins, because the low correlation is also observed even when we consider only monofunctional proteins (*r^2^* = 0.0384% and *p* < 10^−37^ for PIC; *r^2^* = 0.0331% and *p* < 10^−30^ for PEC). Hence, although there is significant non-randomness in protein functions across structural modules, the correspondence between structural modules and functional units is extremely weak in both PIC and PEC networks, especially in the latter.

We also examined the cellular locations of each protein [34] and tested whether members of a structural module tend to be co-localized, as would be expected if structural modules represent functional units. Our results were generally similar to those for functional categories. Although some nonrandom patterns were observed, the correspondence between structural modules and cellular locations is extremely weak in both PIC and PEC networks, especially in the latter (Figure S1).

### Are Structural Modules Evolutionarily Conserved?

If a structurally defined PPI module represents a functional unit, the composition of the module should be evolutionarily conserved. To test this prediction, we applied the same module separation algorithm to the fruit fly (Drosophila melanogaster) PPI network, which was constructed from binary PPIs obtained in high-throughput Y2H experiments [35]. Because the fly data do not contain any interactions inferred from protein complexes, we expect that the fly PPI network behaves more similarly to the yeast PEC network than to the PIC network. We thus examine the evolutionary conservation of modular structures between the yeast PEC network and the fly network.

**Figure 5:**
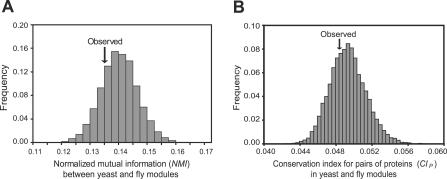
Lack of Evolutionary Conservation between the Yeast and Fruit Fly PPI Modules (A) The observed *NMI* between yeast and fruit fly modules is not significantly different from chance expectation. The bars show the distribution of *NMI* between yeast and fly modules when the yeast modules are randomly separated. (B) The observed *CI_P_* (conservation index for pairs of proteins) between yeast and fruit fly modules is not significantly different from chance expectation. The bars show the distribution of *CI_P_* between yeast and fly modules when the yeast modules are randomly separated.

We separated the fly network into 27 modules, with a modularity of 0.6851 and a scaled modularity of 29 (Table 1). Hence, the scaled modularity of the fly network is comparable to that of the yeast PEC network (27). There are 691 orthologous proteins between the giant component of the yeast PEC network and that of the fly network. We here again use *NMI* to measure the similarity in module compositions between two networks. The *NMI* value between the yeast PEC and fly PPI networks is 0.14. If the modular structures are evolutionary conserved between the two networks, the above *NMI* value should be significantly greater than that between the actual fly network and a randomly separated yeast network. We randomly separated the yeast PEC network into 26 modules by conserving the actual module sizes and then computed *NMI* between the real fly modules and the randomly separated yeast modules. To make this comparison, we repeated this process 10,000 times and obtained the frequency distribution of *NMI* (Figure 5A). The observed *NMI* between the real fly and real yeast networks falls in the central part of the distribution, indicating that the yeast and fly modules are no more similar to each other than by chance (*p* > 0.6) and revealing a complete lack of evolutionary conservation in PPI modules between the two species.

Because modular structures are often hierarchically organized [7], it is possible that a low level of structure is evolutionarily conserved despite the lack of conservation at the whole-module level. Pairwise relationships between proteins represent the lowest possible structure in the PPI network. We invented a conservation index for pairs of proteins (*CI_P_*). Between species X and Y, *CI_P_* is defined as the probability that the Y orthologs of two X proteins belonging to the same module in X also belong to the same module in Y. *CI_P_* is 0.048 between the yeast and fly, which is not significantly different from the expectation derived by comparison of the fly network to a random separation of the yeast network (*p* > 0.6; 10,000 simulations; Figure 5B). Thus, even at the lowest structural level, yeast and fly modules are not evolutionarily conserved. Note that *CI_P_* measures the conservation of co-membership in a module between two proteins, regardless of whether these two proteins interact with each other. *CI_P_* does not measure the conservation of PPIs. If two yeast proteins engage in a PPI and their respective fly orthologs also engage in a PPI, these two PPIs are referred to as orthologous PPIs [36]. Between the yeast PEC and fly PPI networks, there are 45 orthologous PPIs. In comparison, between the fly network and 1,000 randomly rewired yeast networks (with the degree of each node unchanged), there are only 0.58 orthologous PPIs on average (standard deviation = 0.75). Thus, orthologous PPIs are evolutionarily conserved between the two species.

We also examined the evolutionary conservation of structural modules between yeast and the nematode Caenorhabditis elegans. Although the PPI data for C. elegans are highly incomplete, with only 2,387 proteins and 3,825 interactions in the giant component, the results we obtained (Figure S2) are similar to those from the comparison between yeast and fruit fly networks.

### Does Participation in Different Modules Affect the Evolutionary Rate of a Protein?

If structural modules represent functional units, proteins with links to many modules should be evolutionarily more conserved than those with links largely within a module, because multifunctional or pleiotropic proteins tend to be conserved [37,38]. Guimera and Amaral [28] defined the participation coefficient of a node by *PC* = 1 − 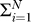 (*k_i_*/*k*)^2^, where *k* is the degree of the node, *k_i_* is the number of links from the node to any nodes in module *I,* and *N* is the total number of modules. A high *PC* indicates that a node participates in the functioning of many modules. These authors found that the propensity for an enzyme gene to be lost during evolution is negatively correlated with the *PC* of the enzyme in the metabolic network [28]. Such an observation strongly suggests that the modular structure in the metabolic network has biological significance. It is therefore useful to examine *PC* for the proteins in the PPI network. It has previously been debated whether the degree of a protein in the PPI network influences its evolutionary rate [39–44]. Because past studies did not exclude PPIs inferred from protein complexes, it is possible that some of previous results were due to artifacts of such inferences. Separate analyses of the PIC and PEC networks may help to answer this question.

We first measured the rate of protein evolution by the number of nonsynonymous nucleotide substitutions per nonsynonymous site (*d*_N_) between orthologous genes of yeast species S. cerevisiae and *S. bayanus*. We chose this species pair because their divergence level is appropriate for obtaining informative and reliable *d*_N_ estimates [45]. We found that the *d*_N_ of a protein is significantly negatively correlated with its total degree in the yeast PIC network (*p* < 0.001; Table 2), but not with its degree in the PEC network (*p* > 0.4). Thus, when protein complexes are not considered, there is no significant correlation between *d*_N_ and degree. When we separated the links of a node into within-module links and between-module links, we found a significant correlation between *d*_N_ and the within-module degree (i.e., the number of within-module links) in the PIC network. This correlation is again absent in the PEC network, suggesting that the correlation between *d*_N_ and within-module degree is largely attributable to protein complexes. In neither the PIC nor the PEC network did we find a significant correlation between *d*_N_ and the between-module degree (i.e., the number of links across modules). Similar results were found between *d*_N_ and *PC* of a protein (Table 2). Furthermore, even when we divided the proteins into different topological roles by their *PCs* and degrees, as was done by Guimera and Amaral for the metabolic network, no significant correlation between these roles and *d*_N_ was observed (Table 2, bottom two rows).

**Table 2:**
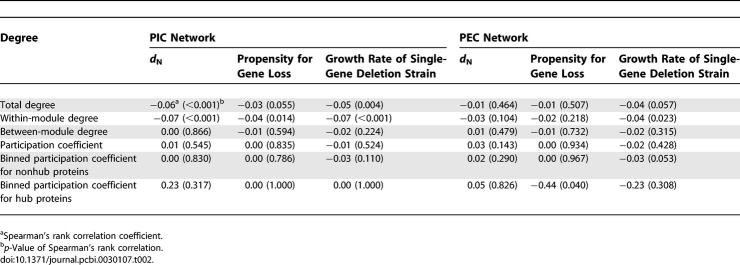
Relationship between the Degree of a Protein and Its Importance to Growth or Evolutionary Rate

We also measured the rate of protein evolution by the propensity for gene loss (*PL*) across 12 fungal species whose draft genome sequences are available. The results obtained for *PL* are qualitatively similar to those for *d*_N_ (Table 2). Taken together, there is no observable impact of the within-module, between-module, or total PPI degree of a protein on its evolutionary rate when protein complexes are excluded. Furthermore, if structural modules correspond to functional units, a protein with higher participation in various modules should be more pleiotropic (or multifunctional) and thus should be more conserved in evolution [37,38]. However, we found no impact of the extent of participation in various modules on the evolutionary rate of a protein. This negative result is consistent with the idea that structural modules do not correspond to functional units.

The growth rate of a yeast strain with a gene deleted can measure the importance of the gene under the tested condition. Growth rate is known to be negatively correlated with the PPI degree of a gene [39,46–48]. We confirmed this result in both PIC and PEC networks, although the significance is only marginal in the latter (Table 2). Interestingly, for both networks, this significance is also found for within-module degrees, but not for between-module degrees. This phenomenon may arise because the between-module degree is often much smaller (mean = 1.04 for PIC and 0.98 for PEC) than the within-module degree (mean = 2.70 for PIC and 2.48 for PEC) and thus contributes less to the total degree of a node. Growth rate also contains the information of gene essentiality, as essential genes have zero growth rates whereas nonessential genes have positive growth rates. Thus, similar results are obtained when we analyze the genes by gene essentiality rather than by growth rate.

### Can Modularity Originate as an Evolutionary Byproduct?

**Figure 6:**
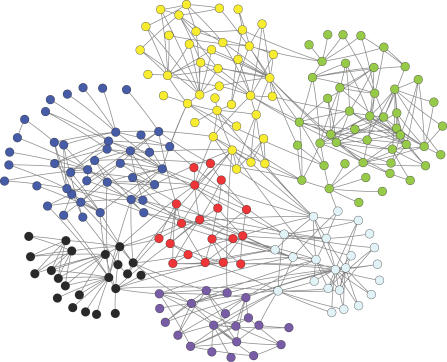
A Random Network Generated by Gene Duplication Followed by Subfunctionalization Shows High Modularity (Modularity = 0.6717, Scaled Modularity = 29) Different colors represent different modules identified by Guimera and Amaral's algorithm [28].

Because both PIC and PEC networks have significantly higher modularity than that of their randomly rewired networks but the identified modules exhibit little biological significance, it is puzzling how the modular structure could have arisen in evolution. Earlier studies suggested that modularity can originate by gene duplication [49,50]. However, in these studies modularity is defined by hierarchical clustering or a clustering coefficient, which lacks an objective function to identify the best module separation and to compute network modularity. We thus conducted computer simulations to examine whether the network modularity as defined in this paper can arise from evolution by gene duplication. Because duplication–divergence models can generate many network features similar to real PPI networks [50,51] and have clear biological bases [52–54], we simulated network growth by a duplication–divergence model starting from a pair of connected nodes. Briefly, at each step, a node (A) is randomly picked and duplicated along with all its edges to generate its paralogous node (A′). We refer to two edges, one from A and the other from A′, as a pair of edges if they both end at the same third node. To simulate functional divergence after gene duplication, we randomly remove one edge from each pair of edges, until A and A′ share 90% of edges. This duplication–divergence process was repeated 300 times to generate a network of 302 nodes. The resulting network has 212 nodes in its giant component (Table S1, first row). We found the modularity and scaled modularity of this simulated network to be 0.6717 and 29, respectively (Figure 6; Table S1). We conducted ten simulation replications, and all cases show similarly high modularity and scaled modularity that are comparable with those of the yeast and fly PPI networks (Table S1). In fact, we found that many different combinations of simulation parameters can give rise to modular networks, and the specific model of evolution by gene duplication (e.g., the subneofunctionalization model [52]) does not appear to matter much to the result of high modularity (unpublished data). Although self-interactions can be biologically important, they are not considered in our simulation because such interactions are disregarded in the module separation algorithm of Guimera and Amaral [28].

## Discussion

In this work, we conducted a comprehensive analysis of modular structures in yeast protein interaction networks. Rather than lumping binary PPIs directly observed in experiments with those indirectly inferred from protein complexes, we separately analyzed the PIC network, which includes inferred binary PPIs, and the PEC network, which excludes inferred binary PPIs. This distinction is necessary because inferences of binary interactions from protein complexes introduce errors to the network structure, which hamper accurate measurement of network modularity. Given that protein complexes likely represent true (functional) modules in the network, the unanswered question is whether the network structure is still modular when the PPIs inferred from protein complexes (~10% in our PIC network) are removed. We found that both PIC and PEC networks are significantly more modular than expected by chance, the scaled modularity of the PIC network is substantively greater than that of the PEC network, and the module compositions of the two networks are significantly different. The latter two results are expected, because the current models for inferring binary PPIs from protein complexes tend to increase modularity. Consistent with these results, we found that the fruit fly PPI network, which is entirely based on experimentally determined binary PPIs, has a comparable scaled modularity to that of the yeast PEC network.

In spite of the presence of significant modularity in the yeast PEC network, the identified structural modules do not appear to correspond to functional units. This is reflected in three analyses. First, for some functional categories, their member genes are distributed randomly among structural modules. Second, for most structural modules, there are no enriched functional categories. Third, for protein pairs, the correlation (*r* ^2^) between co-membership in a module and co-functionality, although significantly greater than zero, is lower than 0.1%. Our results contradict several previous studies which claimed that PPI modules correspond well to functional units [8–16]. This difference is in part owing to the inclusion of protein complexes in these early studies. Furthermore, some studies utilized more than the PPI network topology in separating modules. For example, Tornow and Mewes considered gene co-expression patterns [15]. Although such practices may help identify functional modules, they do not objectively evaluate whether the PPI network itself has a biologically meaningful modular structure. Many studies also suffered from the lack of an efficient algorithm to identify the maximum modularity, resulting in suboptimal modular separations with many small modules. For example, Pereira-Leal and colleagues [13] separated the yeast PPI network into 1,046 modules, with an average size of eight proteins per module. A small module may appear to have a better functional correspondence than a large module, because the chance probability of functional similarity among a few proteins is considerably greater than that among a large number of proteins. Because the module separation algorithm we used here is superior to the earlier algorithms [30], under the same definition of modularity our results are expected to be more reliable than those based on inferior algorithms.

Although many authors have claimed that PPI networks are modular with significant functional correspondence, none have examined the evolutionary conservation of PPI modules. By comparing the yeast PEC network and fly PPI network, we found that PPI modules are not more conserved than the chance expectation at the whole-module level. Furthermore, even at the protein–pair level, the PPI modules are not more conserved than by chance. These findings are consistent with our observation of minimal correspondence between yeast PEC modules and functional units. Interestingly, PPIs are found to be conserved between the yeast and fly, suggesting that the lack of conservation of modules cannot be trivially explained by the lack of conservation of individual interactions in the network.

The participation coefficient of a node measures the extent of the distribution of links from the node to all modules. If PPI modules correspond to functional units, proteins with high participation coefficients should have higher degrees of pleiotropy (or multifunctionality) and be more conserved than those with low participation coefficients, because pleiotropic or multifunctional proteins are known to be evolutionarily conserved [37,38]. This correlation was not observed in either the PIC or PEC network when either *d*_N_ or *PL* was used as a measure of a protein's evolutionary rate. Thus, the results again point to the lack of correspondence between PPI modules and functional units.

Taken together, our analyses strongly suggest that the yeast PEC network has a modular structure, which, nevertheless, lacks detectable biological significance. One may argue that the PEC network actually contains biologically important structural modules, but such modules are difficult to identify due to the incompleteness and inaccuracy of current PPI data. While this possibility cannot be entirely ruled out, we note that the PPI data we used here are generally regarded as of relatively high quality [27]. Furthermore, according to recent estimates, our PPI data should cover 25% to 50% of all PPIs in the yeast interactome [24,55]. Several observations, such as the negative correlation between the growth rate of a single-gene deletion yeast strain and the PPI degree of the gene (Table 2), suggest that the current PPI data contain biologically meaningful signals. An alternative explanation of the PPI modularity that lacks biological significance is that modularity may be an evolutionary byproduct. Inspired by earlier studies [49,50], we demonstrate by computer simulation that a simple model of gene duplication–divergence can generate networks with a scaled modularity comparable to that observed in the yeast and fly PPI networks. This result suggests that the modularity in the PPI networks may indeed have no biological significance and has not been under selection. Because gene duplication is the primary source of new genes and new gene functions [56], our simulation is biologically relevant. It is possible that evolutionary processes other than gene duplication also contributed to the origin of network modularity. For example, if assortative links (i.e., links between nodes of similar degrees) are disfavored, as has been observed in PPI networks [57,58], modularity may arise. PPI networks also have clustering coefficients higher than chance expectation, meaning that two proteins that both interact with the third one also tend to interact with each other [3]. Natural selection for higher clustering coefficients for some nodes of the network may also raise modularity.

It has been intensely debated to whether there is a negative correlation between the PPI degree of a protein and the evolutionary rate (*d*_N_) of the protein [39–44]. We found this correlation to be statistically significant for the PIC network, but not significant for the PEC network. These observations suggest that the significant correlation is simply due to lower evolutionary rates for proteins involved in protein complexes than those not involved in complexes. Our result is consistent with a recent study reporting the lack of a significant correlation when PPIs were curated from literature [39]. Because proteins involved in complexes tend to have exceptionally high degrees as a result of indirect inference of PPIs by the matrix or spoke model, our result is also consistent with the finding that only the most prolific interactors tend to evolve slowly [44]. Recently, Han and colleagues [59] classified hubs (i.e., high-degree nodes) in the PPI network into party hubs and date hubs. The former are those proteins whose interaction partners have similar expression profiles across various conditions, whereas the latter are those whose partners have different expression profiles. Party hubs have been interpreted as proteins that function within a biological process (or a functional module), whereas date hubs are thought to link different functional modules. Fraser reported that party hubs are evolutionarily more conserved than date hubs, and suggested that this pattern may reflect a tendency for evolutionary innovations to occur by altering the proteins and interactions between rather than within modules [60]. A closer examination of the party hubs and their partners reveals that the majority of them form protein complexes, whereas date hubs and their partners do not form complexes. Thus, Fraser's observation is explainable by a lower evolutionary rate of proteins involved in complexes than those not in complexes, without invoking additional evolutionary forces.

PPI networks have been subject to many structural, functional, and evolutionary analyses in the past few years. Our results show that removing a small fraction (~10%) of PPIs that are inferred from protein complexes can have a substantial effect on the analysis. This observation raises a warning about many results regarding PPI networks, because they have usually been based on the PIC network that contains many potentially false PPIs inferred for members of protein complexes. As such false interactions are not randomly distributed in the network, their potential detrimental effect is particularly alarming. The PIC data we used do not contain high-throughput protein complex data such as those in [21,22]. In many PPI databases, such as BIND [61], DIP [62], and the new literature-curated dataset [63], about half or more of the PPIs are inferred from protein complexes. The recent genome-wide surveys of all protein complexes in the yeast added even more complexes to the PPI data. Inclusion of inferred PPIs from these complexes would affect the network structure even more. We caution that use of such PPI data may produce misleading results.

Systems biology is a nascent field with many hopes as well as much hype [64]. It has been of great interest to identify nonrandom topological structures such as motifs and modules in molecular networks [5,28,65]. Such nonrandom patterns are often interpreted as having functional significance and having been particularly favored by natural selection [28,66,67]. While this may be true in many cases, a nonrandom network structure can also originate as a byproduct of other processes without having its own function. Recent studies suggested that motifs in transcriptional regulatory networks do not represent functional units and are not subject to natural selection [68]. Rather, random gene duplication and mutation could give rise to motifs [69]. A recent study even suggested that the high abundance of feed forward loops in regulatory networks could be an evolutionary byproduct [70]. Our results add yet another network structure that is widely believed to be of great biological importance to this growing list of potential evolutionary byproducts. That being said, the modular organization of cellular functions is real, and whether this organization is also an evolutionary byproduct or has been actively selected for remains to be scrutinized.

## Materials and Methods

### The yeast, fly, and nematode PPI networks.

The budding yeast (S. cerevisiae) PPI data were from the MPact dataset [27] of MIPS (ftp://ftpmips.gsf.de/yeast/PPI/PPI_18052006.tab), which contains human-curated high-throughput and small-scale binary interactions directly observed in experiments, as well as binary interactions inferred from high-confidence protein complex data. Only nonself physical interactions were considered. After excluding PPIs involving mitochondrial genes, we built the PPI network named PIC (PPI including protein complexes). The giant component of the PIC network is composed of 3,886 proteins linked by 7,260 nonredundant interactions. To build the PEC (PPI excluding protein *c*omplexes) network, we retained only those binary interactions in the PIC network that had direct experimental evidence. The giant component of the PEC network contains 3,696 proteins linked by 6,403 interactions.

The fruit fly (Drosophila melanogaster) PPI data came from [35] (http://www.bme.jhu.edu/labs/bader/publications/giot_science_2003/flyconf.txt). A moderate confidence level (0.25) was chosen to generate the fly PPI network with a comparable average degree to the yeast PEC network. In total, the giant component of the fly PPI network contains 6,280 proteins linked by 10,210 interactions, all generated by Y2H experiments.

The nematode (C. elegans) PPI data were from [71] (http://vidal.dfci.harvard.edu/interactomedb/WI5.txt). Only the PPIs identified by Y2H experiments are used. In total, the nematode PPI network contains 2,624 proteins and 3,967 interactions, of which 2,387 proteins and 3,825 interactions are in the giant component.

### Functional categories of yeast proteins.

We used the yeast functional annotations in the CYGD database [33] (ftp://ftpmips.gsf.de/yeast/catalogues/funcat/funcat-2.0_data_18052006), considering only the highest level of annotation. Functional categories containing <15 proteins and the category of unknown functions were removed. The cellular localization data for yeast proteins were from [34] (http://yeastgfp.ucsf.edu/allOrfData.txt). Similarly, ambiguous localizations and localizations with <15 proteins were not used.

### Evolutionary conservation of modules and proteins.

The list of orthologous genes between the yeast and fly was provided by He and Zhang [46], who used reciprocal best-hits in BLASTP searches to define gene orthology (E-value cutoff = 10^−10^). The same method was used to identify the yeast and nematode orthologous genes. The *d*_N_ values between *S. cerevisiae* and S. bayanus orthologous genes were computed by a likelihood method and obtained from Zhang and He [45]. We used the parsimony principle to infer the *PL* (i.e., the number of gene loss events) for each of the S. cerevisiae genes throughout the known phylogeny of 12 fungi. The protein sequences predicted from the complete genome sequences of the 12 species were downloaded from ftp://genome-ftp.stanford.edu/pub/yeast/data_download/sequence (S. cerevisiae, S. bayanus, S. paradoxus, and *S. mikatae*), ftp://ftp.ncbi.nih.gov/genomes/Fungi (Candida glabrata, Kluyveromyces lactis, Eremothecium gossypii, Debaryomyces hansenii, and Yarrowia lipolytica), http://www.broad.mit.edu/annotation/genome/neurospora/Home.html (Neurospora crassa), http://www.broad.mit.edu/seq/YeastDuplication (K. waltii), and http://www.sanger.ac.uk/Projects/S_pombe/ (Schizosaccharomyces pombe). A S. cerevisiae gene is considered to be lost in species X if it does not hit any genes in X (Evalue cutoff = 10^−1^) but has a hit in at least one species that is more distantly related to S. cerevisiae than X is related to S. cerevisiae. Here X refers to one of the ten fungi that are neither S. cerevisiae nor *S. pombe,* the latter being the most distantly related species to S. cerevisiae in our study.

The growth rates of the yeast single-gene deletion strains were originally generated by the Stanford Genome Technological Center [72], and we here used the dataset curated and provided by Zhang and He [45].

### Normalized mutual information.

*NMI* was described in detail in [30]. Briefly, let us define the matrix *N,* where each row corresponds to a module in separation X and each column corresponds to a module in separation Y. Each member *N_ij_* in the matrix represents the number of nodes in the *i*th module of X that appear in the *j*th module of Y. The calculation of *NMI* is given by

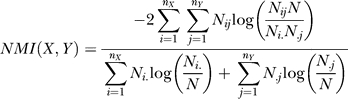
where *n_X_* and *n_Y_* are the number of modules in module separation X and Y, respectively. The sum over row *i* of matrix *N_ij_* is denoted *N_i_*, and the sum over column *j* is denoted *N_j_*. If two module separations are identical, the *NMI* between them reaches the maximum value of 1.

### Data and program availability.

Datasets used in this work and computer programs made for the analyses can be downloaded from http://www.umich.edu/~zhanglab/download.htm.

## Supporting Information

Figure S1 Lack of Obvious Correspondence between Structural Modules and Protein Cellular Locations (A,B) Each cellular location is indicated by a letter (A to U). In parentheses next to the letter is the percentage of proteins in the network that belong to that cellular location. Note that one protein may belong to more than one location. The circles next to the grid show the statistical significance of nonrandom distributions of genes of the same cellular locations across modules. Each small square in the grid shows the statistical significance of enrichment of a particular location in a module. For the circles and squares, significance levels are indicated by different colors.
(C,D) Show the correlation between co-membership in structural modules and co-localization in cellular components for all pairs of proteins in the PIC and PEC networks, respectively. The circle size is proportional to the number of protein pairs. The line shows the linear regression and *r* is the correlation coefficient.
(389 KB PDF)

Figure S2 Lack of Evolutionary Conservation between the Yeast and Nematode PPI Modules (A) The observed *NMI* between yeast and nematode modules is not significantly different from chance expectation. The bars show the distribution of *NMI* between the yeast and nematode modules when the yeast modules are randomly separated.
(B) The observed *CI_P_* (conservation index for pairs of proteins) between yeast and nematode modules is not significantly different from chance expectation. The bars show the distribution of *CI_P_* between the yeast and nematode modules when the yeast modules are randomly separated.
(253 KB PDF)

Table S1 Summary Statistics of the Giant Component in the Random Networks Generated by the Duplication–Divergence Model (75 KB PDF)

## Abbreviations

MIPS: Munich Information Center for Protein Sequences
*NMI*: normalized mutual information
PEC: PPI excluding protein complexes
PIC: PPI including protein complexes
PL: propensity for gene loss
PPI: protein–protein interaction
Y2H: yeast two-hybrid
