# Correction: In Search of the Biological Significance of Modular Structures in Protein Networks

**DOI:** 10.1371/journal.pcbi.0030146

**Published:** 2007-07-27

**Authors:** Zhi Wang, Jianzhi Zhang

In *PLoS Computational Biology*, volume 3, issue 6: 10.1371/journal.pcbi.0030107

In the subsection "Evolutionary conservation of modules and proteins" of the Materials and Methods section, a link was incomplete. The correct link reads:

http://rd.plos.org/10.1371_journal.pcbi.0030107_01_0

