# Supplementary material for: In Search of the Biological Significance of Modular Structures in Protein Networks
Source: PLoS Comput Biol. 2007 Jun 1;3(6):e107. doi: 10.1371/journal.pcbi.0030107 (PMC1885274; doi:10.1371/journal.pcbi.0030107)
Supplement: Supplementary file 1 [file pcbi.0030107.sg001.pdf]

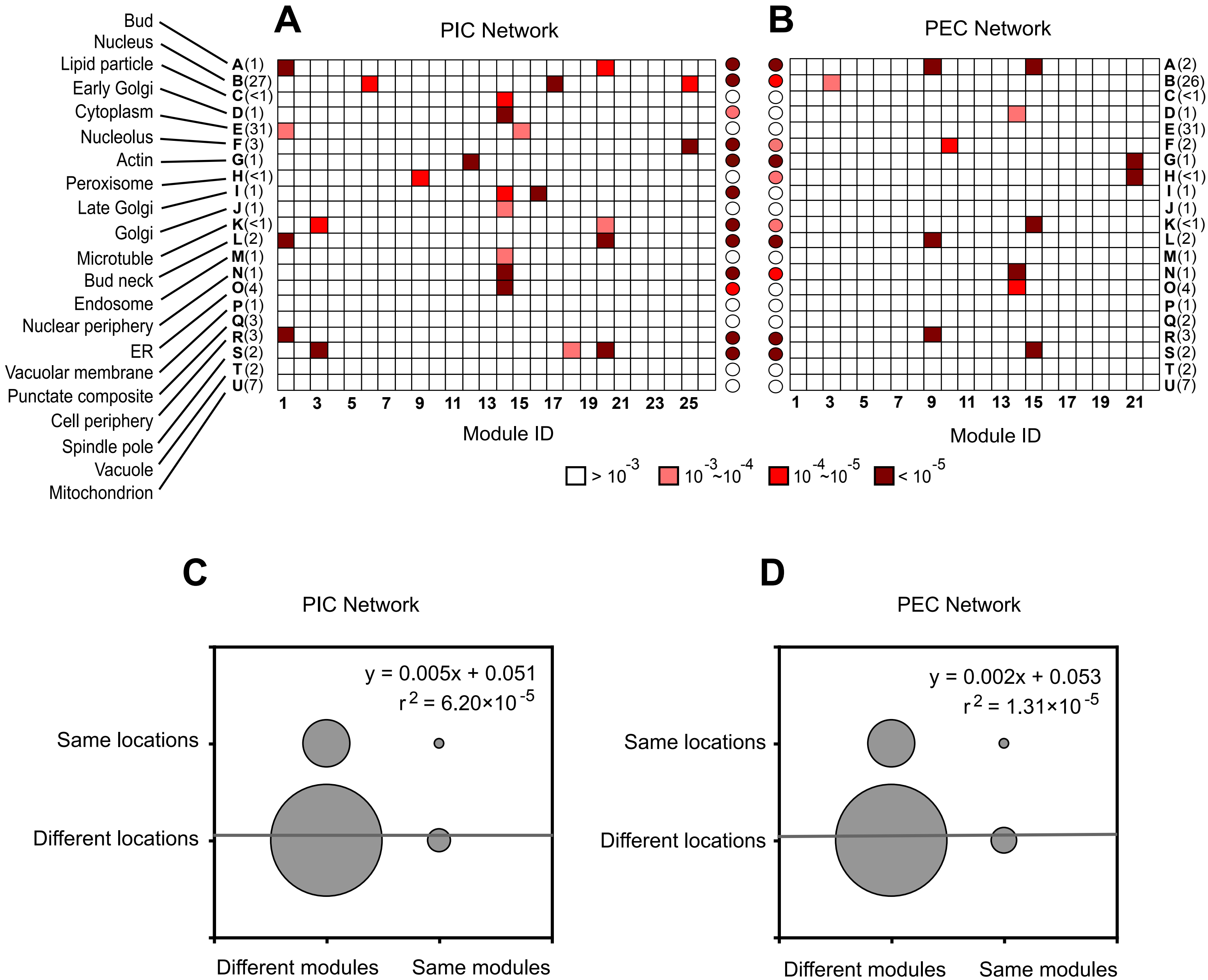

A
PIC Network
B
PEC Network
Bud
Nucleus
Lipid particle
Early Golgi
Cytoplasm
Nucleolus
Actin
Peroxisome
Late Golgi
Golgi
Microtubule
Bud neck
Endosome
Nuclear periphery
ER
Vacuolar membrane
Punctate composite
Cell periphery
Spindle pole
Vacuole
Mitochondrion
A(1) B(27) C(<1) D(1) E(31) F(3) G(1) H(<1) I(1) J(1) K(<1) L(2) M(1) N(1) O(4) P(1) Q(3) R(3) S(2) T(2) U(7)
A(2) B(26) C(<1) D(1) E(31) F(2) G(1) H(<1) I(1) J(1) K(<1) L(2) M(1) N(1) O(4) P(1) Q(2) R(3) S(2) T(2) U(7)
1 3 5 7 9 11 13 15 17 19 21 23 25
1 3 5 7 9 11 13 15 17 19 21
Module ID
$> 10^{-3}$
$10^{-3} \sim 10^{-4}$
$10^{-4} \sim 10^{-5}$
$< 10^{-5}$
C
PIC Network
$y = 0.005x + 0.051$
$r^2 = 6.20 \times 10^{-5}$
D
PEC Network
$y = 0.002x + 0.053$
$r^2 = 1.31 \times 10^{-5}$
Same locations
Different locations
Different modules
Same modules
