# Supplementary material for: In Search of the Biological Significance of Modular Structures in Protein Networks
Source: PLoS Comput Biol. 2007 Jun 1;3(6):e107. doi: 10.1371/journal.pcbi.0030107 (PMC1885274; doi:10.1371/journal.pcbi.0030107)
Supplement: Supplementary file 2 [file pcbi.0030107.sg002.pdf]

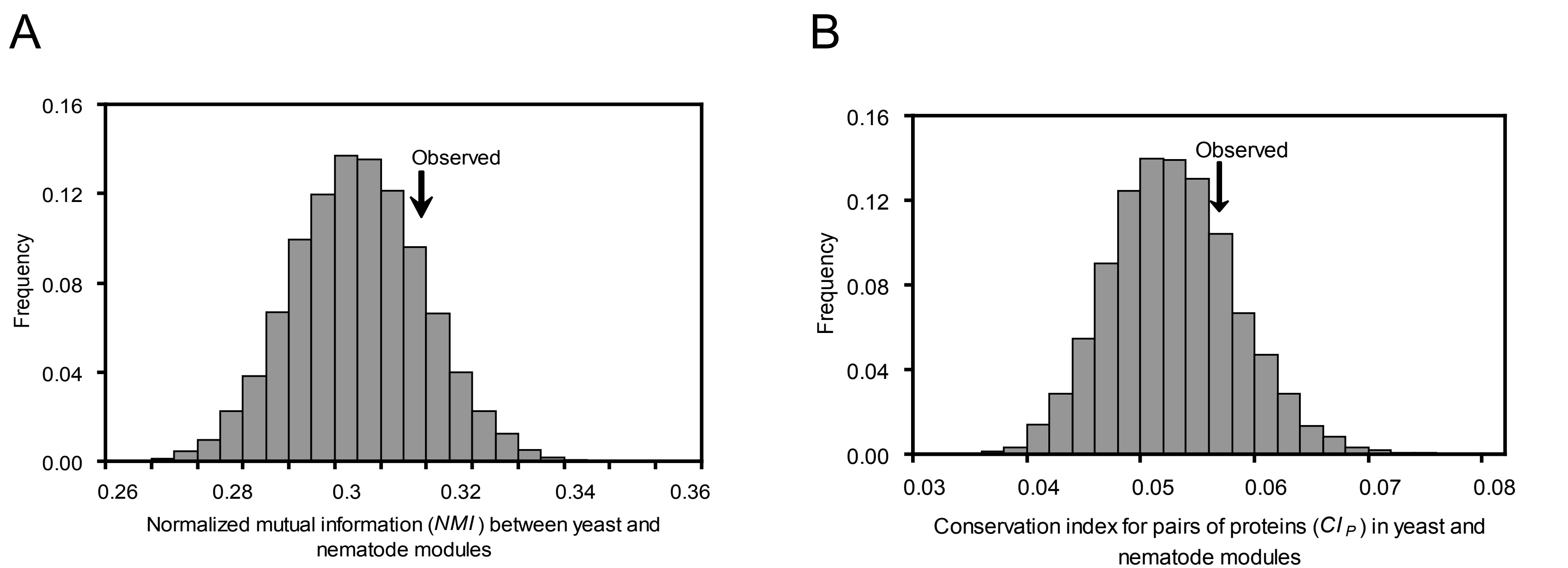
A
0.16
0.12
0.08
0.04
0.00
Frequency
Observed
0.26
0.28
0.3
0.32
0.34
0.36
Normalized mutual information (NMI) between yeast and nematode modules
B
0.16
0.12
0.08
0.04
0.00
Frequency
Observed
0.03
0.04
0.05
0.06
0.07
0.08
Conservation index for pairs of proteins (CI P) in yeast and nematode modules
