# Supplementary material for: In Search of the Biological Significance of Modular Structures in Protein Networks
Source: PLoS Comput Biol. 2007 Jun 1;3(6):e107. doi: 10.1371/journal.pcbi.0030107 (PMC1885274; doi:10.1371/journal.pcbi.0030107)
Supplement: Supplementary file 3 [file pcbi.0030107.st001.pdf]

Table S1. Summary statistics of the giant component in the random networks generated by gene duplication followed by subfunctionalization.

Networks	No. of nodes	No. of interactions	Modularity	Mean modularity of randomly rewired networks	Standard deviation of modularity in randomly rewired networks	Scaled modularity
1	212	1028	0.6717	0.4485	0.0077	29
2	186	1088	0.6001	0.3936	0.0063	33
3	155	864	0.5884	0.3979	0.0074	26
4	136	634	0.5917	0.4402	0.0091	17
5	205	1032	0.6193	0.4289	0.0064	30
6	185	860	0.6498	0.4605	0.0077	25
7	221	1194	0.6466	0.4208	0.0072	32
8	146	760	0.6525	0.4247	0.0093	25
9	148	754	0.5934	0.4044	0.0067	28
10	118	564	0.6164	0.4202	0.0091	22
Average	171.2	877.8	0.6230	0.4240	0.0077	26
